# Emulsion Polymerizations for a Sustainable Preparation of Efficient TEMPO‐based Electrodes

**DOI:** 10.1002/cssc.202002251

**Published:** 2020-11-20

**Authors:** Simon Muench, Patrick Gerlach, René Burges, Maria Strumpf, Stephanie Hoeppener, Andreas Wild, Alexandra Lex‐Balducci, Andrea Balducci, Johannes C. Brendel, Ulrich S. Schubert

**Affiliations:** ^1^ Laboratory of Organic and Macromolecular Chemistry (IOMC) Friedrich Schiller University Jena Humboldtstr. 10 07743 Jena Germany; ^2^ Center for Energy and Environmental Chemistry Jena (CEEC Jena) Friedrich Schiller University Jena Philosophenweg 7a 07743 Jena Germany; ^3^ Institute for Technical Chemistry and Environmental Chemistry Friedrich Schiller University Jena Philosophenweg 7a 07743 Jena Germany; ^4^ Jena Center for Soft Matter (JCSM) Friedrich Schiller University Jena Philosophenweg 7 07743 Jena Germany; ^5^ Evonik Operations GmbH Research, Development & Innovation Paul-Baumann-Straße 1 45772 Marl Germany

**Keywords:** composite materials, electrode materials, emulsion polymerization, energy storage, organic battery

## Abstract

Organic polymer‐based batteries represent a promising alternative to present‐day metal‐based systems and a valuable step toward printable and customizable energy storage devices. However, most scientific work is focussed on the development of new redox‐active organic materials, while straightforward manufacturing and sustainable materials and production will be a necessary key for the transformation to mass market applications. Here, a new synthetic approach for 2,2,6,6‐tetramethyl‐4‐piperinidyl‐*N*‐oxyl (TEMPO)‐based polymer particles by emulsion polymerization and their electrochemical investigation are reported. The developed emulsion polymerization protocol based on an aqueous reaction medium allowed the sustainable synthesis of a redox‐active electrode material, combined with simple variation of the polymer particle size, which enabled the preparation of nanoparticles from 35 to 138 nm. Their application in cell experiments revealed a significant effect of the size of the active‐polymer particles on the performance of poly(2,2,6,6‐tetramethyl‐4‐piperinidyl‐*N*‐oxyl methacrylate) (PTMA)‐based electrodes. In particular rate capabilities were found to be reduced with larger diameters. Nevertheless, all cells based on the different particles revealed the ability to recover from temporary capacity loss due to application of very high charge/discharge rates.

## Graphical abstract

**Sustainable and efficient organic electrode**: A new synthetic approach for polymers for organic batteries includes an emulsion polymerization with adjustable particle sizes in aqueous dispersions and allows the sustainable manufacturing of active materials and composite electrodes. The electrochemical investigation shows that the influence of particle sizes and the resulting morphologies of composite films on the cell performance is as important as the active material itself.

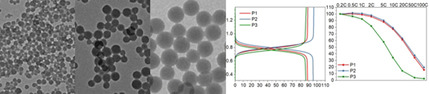

## 1 Introduction

Organic batteries represent a promising element for the future of mobile energy supply and feature several advantages compared to present‐day metal‐based battery systems.^[1]^ They can be produced by straightforward manufacturing techniques, such as printing or roll‐to‐roll processing, which allows the production of large quantities at low costs.^[2]^ Moreover, these techniques enable customizable designs to meet the requirements of the large variety of gadgets for the upcoming internet of things and allow mechanically flexible devices.^[3]^ Their compounds can prospectively be prepared from renewable resources and at the end of their lifetime they can be disposed by incineration without toxic leftovers like metal oxides.^[4]^

Much scientific work on organic electrode materials was conducted over the last years.^[5]^ However, the transition of this new technology into mass market devices is still in its infancy. To facilitate this transition, two important aspects should be in the focus of future developments: competitive prices and the sustainability of containing materials and their production. The mass production of polymers bears the opportunity to significantly decrease the costs. But many polymerization techniques include expensive and toxic reactants as well as solvents. Therefore, a straightforward manufacturing technique is required that circumvents these issues. Here, the application of emulsion polymerization techniques can be of great benefit. Emulsion polymerizations are well established in industrial polymer synthesis (e. g., for paints and coatings) due to several explicit advantages. They combine the synthesis of hydrophobic polymer particles with a water‐based production technique and, thus, no organic solvents are required. They are easily scalable, reproducible, and reveal fast polymerization kinetics due to the suppression of termination reactions.^[6]^ Furthermore, no elaborate purification is necessary, as the insoluble polymer particles can be isolated by filtration and the dried particles can be utilized in battery manufacturing without further processing steps like milling.

Recently, catechol‐based polymer nanoparticles were reported as organic electrode materials for lithium batteries, while redox‐active colloids were investigated in suspension‐based flow batteries.^[7]^ However, the here applied poly(2,2,6,6‐tetramethyl‐4‐piperinidyl‐*N*‐oxyl methacrylate) (PTMA) represents one of the most promising candidates for active materials for organic batteries. It is synthesized by polymerization of 2,2,6,6‐tetramethyl‐4‐piperidyl methacrylate (TMPMA) and subsequent oxidation to the 2,2,6,6‐tetramethyl‐4‐piperinidyl‐*N*‐oxyl (TEMPO) free radical‐containing PTMA.^[8]^ It undergoes quasi‐reversible oxidation and reduction and is suitable as an active cathode material with high rate capability.^[9]^ However, not only the redox‐characteristics of the materials itself but also the morphology and particle structure have an influence on the battery performance.^[10]^ A high particle surface is supposed to improve the migration of counter ions from the electrolyte in and out of the active material during the charge and discharge processes. Furthermore, a good processability is important, while maintaining insolubility to prevent the dissolution of active material into the electrolyte. Up to now, PTMA is commonly synthesized in organic solvent‐based polymerizations that require further processing steps, like milling, to receive suitable particle sizes. In emulsion polymerizations, however, the particle size can be easily adjusted by variation of the surfactant concentration.

In this work we report that classical polymerization techniques can be applied to yield aqueous dispersions of PTMA active material with the advantage of adjustable particle sizes between 35 to 138 nm. Consequently, the influence of the particle size on the cell performance of PTMA‐containing composite electrodes is investigated. Furthermore, all components, such as cross‐linker, surfactant, initiator, and oxidant were selected in view to sustainable synthesis on an industrial scale.

## 2 Results and Discussion

### 2.1 Polymer particle synthesis

**Scheme 1:**
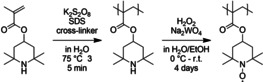
Schematic representation of the polymerization of TMPMA and the oxidation of poly(TMPMA) to PTMA.

PTMA is synthesized in two steps starting from commercial materials: First, the polymerization of TMPMA, and second, the oxidation to PTMA (Scheme 1). The polymerization is often carried out applying a living polymerization technique [reversible addition–fragmentation chain‐transfer (RAFT), atom transfer radical polymerization (ATRP), etc.] in scientific studies.^[11]^ However, since the dispersity is less important for an application in batteries, in particular for cross‐linked particles, a free‐radical polymerization is mainly applied and the initiation technique of choice for an industrial application. For the development of a protocol for the emulsion polymerization of TMPMA we adapted standard textbook reaction conditions.^[12]^ Sodium dodecyl sulfate (SDS) was chosen as surfactant and applied in different concentrations, whereas K_2_S_2_O_8_ was used as initiator. For cross‐linked particles, bifunctional methacrylate‐based cross‐linkers were applied.

A special feature of this reaction, which is usually performed with liquid hydrophobic monomers, is that the monomer TMPMA is solid under ambient conditions and has to be molten before polymerization to form a dispersion. Therefore, the mixture of SDS and TMPMA in water was heated to 75 °C and stirred for 45 min until a uniform dispersion was formed. After the addition of a cross‐linker, the polymerization was initiated by K_2_S_2_O_8_ at 75 °C. The fast reaction rates of emulsion polymerizations enabled short reaction times of 35 min. During the reaction, a color change from pale rose‐red to light blue was observed, caused by light scattering of the nm‐scaled particles.

**Figure 1:**
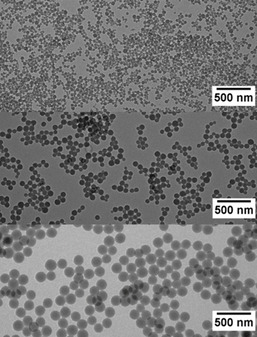
TEM images of poly(TMPMA) P1 (top, 35±11 nm), P2 (middle, 73±12 nm), and P3 (bottom, 138±13 nm).

A polymerization without cross‐linker and a surfactant concentration of 3.6 mol % SDS resulted in particles with a diameter of 31±9 nm (Figure S1). However, these non‐cross‐linked particles are soluble in organic solvents. This might be beneficial for the electrode manufacturing and an efficient coating of conductive additive by the active material, but it also leads to a solubility of the active material in common battery electrolytes, causing a capacity loss and self‐discharge by shuttling of the charged polymer. As a consequence, different cross‐linkers were tested to obtain insoluble polymer particles. It is expected that the length of the spacer influences the efficiency of cross‐linking due to a higher flexibility. Therefore, two cross‐linkers with different spacer length, triethylene glycol dimethacrylate (TEG‐DMA) and ethylene glycol dimethacrylate (EG‐DMA), were applied. The use of 3 mol % TEG‐DMA as cross‐linker resulted in a suspension of polymer particles. However, TEM images (Figure S2) revealed an undesired linking of the particles to clusters and the formation of ill‐defined particles. This might be explained by the high affinity of this cross‐linker to water and a resulting accumulation at the surface, followed by linking of the particles. Therefore, a cross‐linker with a shorter ethylene glycol spacer was chosen. In the case of 3 mol % EG‐DMA, well defined nanoparticles were formed as suspension (Figure 1).

**Figure 2:**
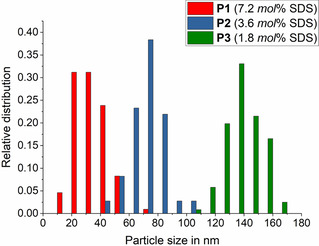
Distribution of poly(TMPMA) particle sizes determined from TEM images.

The ratio of surfactant to monomer in an emulsion polymerization is typically used to vary the size of the resulting particles. To investigate the influence of the surfactant on the particle size, the concentration of SDS was varied. As expected, an increase in the surfactant concentration leads to a higher concentration of micelles, which serve as nuclei for the polymerization. Consequently, the constant amount of monomer is distributed over a larger number of polymer particles, resulting in a reduced size. While a concentration of 7.2 mol % SDS (P1) leads to an average particle size of 35 nm, 3.6 mol % SDS (P2) yield 73 nm particles, and 1.8 mol % (P3) leads to particles of 138 nm (Table 1). The standard deviations of 11–13 nm reveal rather narrow size distributions (Figure 2).

**Table 1:** Applied surfactant concentrations and resulting particle characteristics.

Polymer	SDS conc. [mol %]	Particle size^[a]^ [nm]	Degree of oxidation^[b]^ [%]
P1	7.2	35±11	70±10
P2	3.6	73±12	73±8
P3	1.8	138±13	71±2

[a] Particle sizes determined from TEM images of poly(TMPMA) before oxidation. [b] Determined by triple determination of ESR spectroscopy.

The precursor polymers were subsequently oxidized to yield PTMA. The oxidation of the poly(TMPMA) to PTMA was performed in a polymer‐analogous reaction with H_2_O_2_ catalyzed by Na_2_WO_4_ in an aqueous‐based reaction mixture. Because poly(TMPMA) is not soluble in water and the polymer particles are not swollen, the reaction sites inside the particles are difficult to access for the oxidant, which limits the conversion. As a consequence, mixtures of an organic solvent and water were applied to enhance the accessibility for the oxidation agent. Ethanol was chosen as an environmentally friendly and cheap co‐solvent. The addition leads to an enhanced swelling of the polymer and, consequently, to a more efficient oxidation. Furthermore, the reaction temperature has an influence. It was found that a low temperature of 0 °C is beneficial.

SEM images of the PTMA particles after washing and drying show an aggregation of the oxidized particles (Figure S3). However, the initial particles can still be identified in these aggregates, and their sizes are maintained after the oxidation.

The degrees of oxidation were determined by electron spin resonance (ESR) spectroscopy of the dried PTMA‐particles and range from 70 to 73 % (Table 1). The difference between the samples is well within the error range, which shows that the particle size has no significant influence on the degree of oxidation. However, it should be taken into consideration that the spin counting by ESR represents no absolute method, and the quantitative results are strongly influenced by several parameters, such as the morphology of the analyte. A sample of linear PTMA polymer with known radical content (determined by titration) was chosen as calibration standard to mimic the nature of the samples as closely as possible. Nevertheless, the determined radical contents allow the comparison of similar samples with each other (in this case the nanoparticles P1–P3), but should be considered with caution with regard to absolute values.

### 2.2 Electrochemical investigations

In order to compare and understand the influence of the three different particle sizes on the electrochemical behavior of PTMA‐based electrodes, composite electrodes containing the polymers P1, P2, and P3 as active material were prepared (see Figure S4 for SEM images). Carboxymethyl cellulose was chosen as binder for a sustainable composite fabrication, which is not only bio‐based but, furthermore, allows the application of water as environmentally friendly alternative to harmful organic solvents. The electrodes were tested in combination with an oversized self‐standing activated carbon‐based counter electrode and 1 m 1‐butyl‐1‐methylpyrrolidinium *bis*(trifluoromethanesulfonyl)imide (Pyr_14_TFSI) in propylene carbonate (PC) as electrolyte. At room temperature, this electrolyte displays a viscosity of 5.1 mPa s^−1^ and an ionic conductivity of 7.3 mS cm^−1^.^[13]^ In the past, it has been used as electrolyte for electrochemical double layer capacitors (EDLCs), as well as for PTMA‐based devices.[^13^, ^14^]

**Figure 3:**
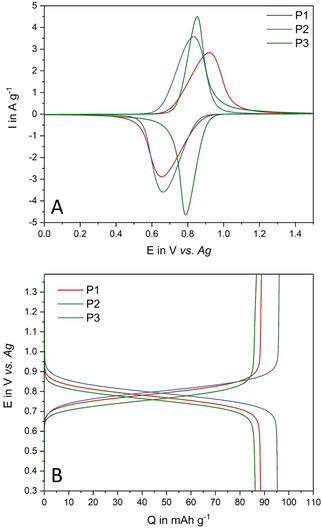
A) Cyclic voltammograms at a scan rate of 2 mV s^−1^ and B) voltage profile at a current density of 1C of the electrodes with P1, P2, and P3 in 1 m Pyr_14_TFSI in PC.

Figure 3 shows the electrochemical performance of PTMA electrodes containing P1, P2, and P3 (for better readability these will in the following be simply indicated as P1, P2, and P3, respectively) after 5 h of open circuit voltage (OCV). As shown in cyclic voltammetry (CV) measurements (Figure 3 A), all electrodes, independently from the particle size of the active material, displayed a reversible redox behavior. However, differences in the shape of the resulting curves were found.

While the average voltages of the redox peaks are similar for all particle sizes (≈0.75 V vs. Ag), P1 displays a large voltage split between oxidation and reduction peaks and the lowest current of the three electrodes of approximately 2.8 A g^−1^. P2 shows a smaller shift and a higher current, while P3 completes this trend with the closest peak split and the highest measured current of 4.5 A g^−1^. The measured potentials comply with the expected value for TEMPO radicals. The voltage profiles of the investigated electrodes at a current density of 1 C (Figure 3B) reveal similar distinct redox plateaus for all polymers with potentials near the values obtained in the CV measurements. While P1 and P3 show nearly the same specific capacity of approximately 87 mAh g^−1^, P2 reveals a higher capacity of 96 mAh g^−1^.

**Figure 4:**
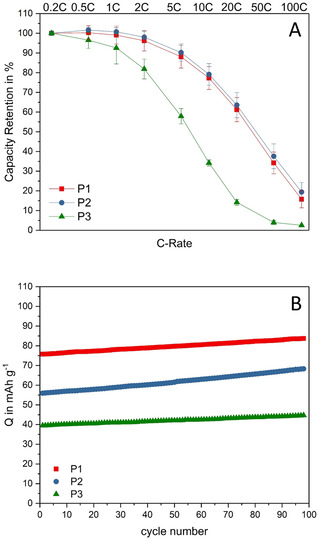
A) Rate capability of the electrodes with P1, P2, and P3 in 1 m Pyr_14_TFSI in PC at 0.2–100C; exemplary cell of triple determination with corresponding error bars. B) Cycling stability of the electrodes at 1C.

Figure 4 A shows the rate capability of the three PTMA‐based electrodes (in relation to their maximum capacity reached at 0.2 C). The depicted results are exemplary cells from triple determinations. Among the electrodes, P1 and P2 reveal nearly the same high capacity retentions. Up to 1 C, the electrodes are able to keep their initial capacities. At 10 C, the electrodes retain approximately 80 %, and at 100 C they still deliver 15–20 % of the initial capacity. The performance of P3, however, is much more affected at higher current densities. While the capacity retention at 10 C is only 34 %, P3 is not able to retain almost any capacity at 100 C. Due to the larger particle size, the accessibility of the inner redox‐active moieties might be impeded, which limits the performance. The overall surface area is lower compared to the bulk volume, resulting in increased diffusion length for the counter ions. Therefore, charge propagation and counter ion mobility are inhibited, which affects the rate capability. The corresponding deviations confirm the good reproducibility of this experiment.

Immediately after the rate capability tests, the cycling stability of the electrodes at 1 C was investigated (Figure 4B). In this experiment, all electrodes display significantly lower specific capacities, compared to the previous test at 1 C (Figure 3B). P1 displays an initial capacity of 76 mAh g^−1^, which recovers to 84 mAh g^−1^ after 100 cycles. In comparison, P2 lost more of its initial capacity and starts at 56 mAh g^−1^, increasing to 68 mAh g^−1^ upon cycling. P3 follows the trend of higher capacity loss with larger particle size and displays a rather low initial capacity of 40 mAh g^−1^, however, also improves to 45 mAh g^−1^ after 100 cycles. The significantly decreased capacities are an effect of the high stress for the electrode materials during rate capability tests at up to 100 C, which seems to be more prominent for larger PTMA particles. Conformational changes during the fast charging processes and the limited diffusion of the counter ions can lead to clocking of pores and diffusion pathways. Often, these effects are irreversible; however, P1, P2, and P3 reveal the ability to slowly recover their capacity from cycle to cycle, which was still ongoing at the end of this test (100 cycles).

**Figure 5:**
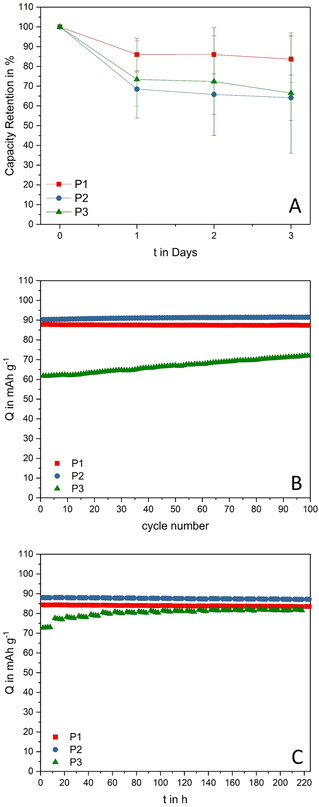
A) Capacity retention of P1, P2, and P3 after three days of self‐discharge; exemplary cell of triple determination with corresponding error bars. B) Cycling stability at 1C after self‐discharge tests. C) Specific capacity of P1, P2, and P3 during float tests performed at 1.1 V vs. Ag for 220 h.

Figure 5 A shows the capacity retention of the investigated electrodes after 1, 2, and 3 days of self‐discharge. This latter process, which can be caused by redox shuttling of dissolved active material in the electrolyte, is known to occur in PTMA‐based electrodes, affecting their behavior when used in energy storage devices. Overall, all electrodes display a rather low self‐discharge, and they are stabilizing their behavior after one day. Interestingly, electrode P1, featuring the smallest particles and the highest capacity after cycling, revealed the lowest capacity loss of 16 % after three days. P2 and P3 displayed a very similar behavior but only maintained approximately 65 % of their initial capacity after three days. However, in this experiment the standard deviation of the triple determination is higher, and, therefore, the obtained values do not differ significantly. Nevertheless, it proves that the crosslinking of the polymers in the particles seems to be sufficient to prevent a severe capacity loss independent of the particle size.

Subsequently, the cycling performance of the electrodes was tested again (Figure 5B). P1 and P2 display a very stable cycling behavior without notable degradation at capacities close to those observed in the first galvanostatic test (Figure 3B). Only P3 shows a lower capacity but still recovers continuously during the cycling process, slowly approaching the initial specific capacity. These results further indicate the ability of all applied polymers to recover from the performance losses caused by the very high current rates.

This observation is further emphasized in float tests. For these tests, the electrode potential is held at a certain value over a long period of time (in this case 220 h). During float tests performed at 1.1 V vs. Ag (Figure 5 C), the electrodes P1 and P2, which already started close to their maximum capacity values, again display a very stable behavior. P3, however, reveals a continuing recovery of capacity. At the end of this test, P1, P2, and P3 deliver capacities of 84, 87, and 81 mAh g^−1^, respectively. This shows that, under prolonged charging conditions, the observed capacity loss is reversible for all tested PTMA‐based electrodes. This recovery requires far more cycles in case of the largest particles (P3), which suggests that conformational changes are involved in the process to restore the accessibility of the inner redox‐active moieties. This behavior is in good agreement with the rapid capacity loss at increasing C‐rates. Taking these results into account, the particle size of the active material does not significantly affect the maximum reachable capacity. However, it considerably influences the electrochemical performance of PTMA‐based electrodes in terms of capacity retention, self‐discharge, and the ability to recover from temporary capacity loss.

## 3 Conclusions

A new synthesis technique for the sustainable preparation of redox‐active poly(2,2,6,6‐tetramethyl‐4‐piperinidyl‐*N*‐oxyl methacrylate) (PTMA) particles for organic batteries by emulsion polymerization with adjustable particle sizes was developed. It could be shown that such processes can be applied in the sustainable manufacturing of battery electrodes, without further processing steps like milling, which represents a valuable step for this field of technology. Polymer particles with three different sizes in the nanometer range were synthesized by an industrially applicable emulsion polymerization process, subsequently oxidized in an aqueous based process, and finally applied in water processed composite electrodes. Overall, the presented processes circumvent the use of any hazardous organic solvents. It could be demonstrated that the developed synthesis protocol reveals the general features of emulsion polymerizations, including short reaction times and variable particle sizes, which could easily be adjusted by the surfactant concentration. Moreover, all materials were chosen to enable a production in a sustainable manner and the applied emulsion polymerization technique enables an easy upscaling without the need for post‐processing.

All investigated electrodes revealed similar high capacities of approximately 90 mAh g^−1^ with the medium‐sized particles (P2) giving slightly higher values. However, the effect of the particle size becomes apparent considering the rate performance tests. P1 and P2 containing the smaller particles featured the highest rate capability (80 % capacity retention up to 10 C), while the largest particles (P3) result in a significant drop (<60 % capacity) at 5 C already. Overall, all tested polymers lost a significant amount of capacity after the application of very high current densities (>20 C). The capacity loss is significantly more pronounced with increasing particle size, since the low surface area and longer diffusion pathways of larger particles limit the accessibility of the redox‐active sites for electrolyte ions. However, subsequently conducted cycling experiments as well as float tests revealed that these capacity losses are reversible, and all investigated electrodes are able to gradually recover their performance.

These findings underline the importance of a profound understanding and optimization of organic battery electrodes on the basis of numerous parameters, including the materials themselves, their morphology and particle sizes. Charge propagation and counter ion mobility are critical. However, the developed preparation protocol opens up a pathway to a sustainable and economical large‐scale production of such electrodes with competitive quality.

## Experimental Section

**Materials**: TMPMA (TCI), SDS, K_2_S_2_O_8_ (Sigma Aldrich), EG‐DMA (Fluka), TEG‐DMA, H_2_O_2_ (Carl Roth), EDTA (Laborchemie Apolda), Na_2_WO_4_ ⋅ 2H_2_O (Acros), SuperP® (Alfa Aesar), carboxymethyl cellulose (Sigma Aldrich), DLC Super 30® (Norit), Super C65® (Imerys), polytetrafluoroethylene (PTFE, Sigma Aldrich), and Pyr_14_TFSI (Iolitec) were purchased from commercial sources and, unless noted otherwise, used as received.

**Typical emulsion polymerization**: TMPMA (31.5 g, 139.8 mmol) and SDS (1470 mg, 5.09 mmol) were dissolved in 420 mL water and deoxygenated for 30 min. The mixture was stirred and heated to 75 °C for 45 min until a uniform emulsion was formed. EG‐DMA (790 μL, 832.5 mg, 4.20 mmol) and a deoxygenated solution of K_2_S_2_O_8_ (523.5 mg, 1.94 mmol) in 15 mL water were added. The mixture was stirred for 35 min at 75 °C, during which the color changed from pale rose‐red to light blue. After cooling to r.t., the reaction mixture was lyophilized to yield 34 g of white powdered product.

**Typical oxidation procedure**: The prepared polymer (4.0 g, 17.75 mmol) was redispersed in 20 mL water and allowed to swell for 30 min. 44.8 mL ethanol were added and the mixture was cooled to 0 °C while stirred. 1 equiv. of H_2_O_2_ (30 % in water, 1.8 mL) was added over the course of 1 h. Afterwards, first ethylenediaminetetraacetic acid (EDTA, 45.3 mg, 0.16 mmol) and subsequently Na_2_WO_4_ ⋅ 2H_2_O (131.1 mg, 0.40 mmol) were added. 2 equiv. of H_2_O_2_ (30 % in water, 3.6 mL) were added over the course of 2 h. After additional 1 h of stirring at 0 °C, 3 equiv. of H_2_O_2_ (50 % in water, 3.0 mL) were added over the course of 1 h, the mixture was stirred for 20 h while slowly warmed to r.t. Again, 3 equiv. of H_2_O_2_ (50 % in water, 3.0 mL) were added and the mixture was stirred for further 72 h. Afterwards, it was heated to 45 °C for 2 h to decompose excess H_2_O_2_ and separated on a POR4 filter. Drying in vacuum yielded 3.6 g of pale rose‐red product. The radical content was determined from the mean values derived from the ESR spectra of three samples per compound using an EMXmicro CW‐EPR spectrometer (Bruker). A known PTMA polymer (radical content of 80 %, determined by redox titration) was used as a reference.

**Electron microscopy**: TEM investigations were performed on a Tecnai G^2^20 (FEI) transmission electron microscope operated at an acceleration voltage of 200 kV. 15 μL of the nanoparticle suspension was blotted onto a carbon coated TEM grid, which was cleaned in an Ar plasma for 1 min prior to use. This step also renders the grid hydrophilic. Excess solution was removed with a filter paper (Whatman No.1). Images were acquired with an Olympus MegaView (OSIS). Contrast enhancement and analysis of the particle sizes is based on ImageJ. SEM images were acquired with a Zeiss Sigma VP at an acceleration voltage of 6 kV for particle evaluation (Figure S3) and 2 kV for the investigation of the morphology of the electrodes (Figure S4). Images were mainly acquired with the SE or the In‐lens detector. Samples were investigated without any additional conductive coating.

**Electrode manufacturing**: PTMA‐based electrodes were prepared by mixing PTMA, SuperP® carbon black as conductive agent, and carboxymethyl cellulose as binder in a mass ration of 60 : 35 : 5 in water with a lab dissolver (Dispermat®, VMA‐GETZMANN). The water‐based slurry was casted with a doctor blade (250 μm blade gap) on an aluminum current collector (pre‐treated with aqueous KOH) and dried overnight at r.t. and for additional 2 h in a vacuum oven at 40 °C. Electrodes were used with an area of 1.13 cm^2^. Therewith the average mass loading of active material on each electrode resulted to 1.28 mg. Oversized free‐standing activated carbon‐based electrodes, which have been used as counter electrodes, were prepared with DLC Super 30® activated carbon, Super C65® as conductive agent, and PTFE as binder in the mass ratio of 85 : 10 : 5. The electrodes displayed an average mass loading of 45 mg and an area of 1.13 cm^2^.

**Electrolyte preparation**: The used electrolyte was prepared by dissolving 1 m Pyr_14_TFSI in dry PC in a glovebox (LabMaster, MBRAUN) with an argon atmosphere (<0.1 ppm water and oxygen). The viscosity of the electrolyte was measured with a rheometer (MCR 102, Anton Paar) using a shear rate of 1000 s^−1^. The conductivity was determined by impedance spectroscopy using a Single Potentiostat (SP150, Biologic Science Instruments).

**Electrochemical Characterization**: All electrochemical measurements were conducted using a VMP multichannel potenstiostatic‐galvanostatic workstation (VMP 3, Biologic Science Instruments) at r.t. The electrodes were applied in a T‐shaped Swagelok‐type 3‐electrode cell setup, with the PTMA composite electrode (containing P1, P2, or P3) as working electrode, an oversized self‐standing carbon counter electrode, and a silver wire as a quasi‐reference. Glass fiber (Whatman®) was used as separator, which was drenched with 160 μL of 1 m Pyr_14_TFSI in PC. Before the first electrochemical measurements, all cells were set to a 5 h OCV step, in order to bring the freshly assembled cells in an electrochemical equilibrium. CV was performed using a scan rate of 2 mV s^−1^. Galvanostatic charge–discharge cycling was carried out between 0.3 and 1.4 V vs. Ag with current densities ranging from 0.2 to 100 C (1 C was defined considering the theoretical capacity of PTMA, 111 mAh g^−1^). The self‐discharge of PTMA‐based electrodes was recorded by fully charging (at 1.4 V vs. Ag) the electrodes at 1 C and monitoring the residual stored charge of the active material by galvanostatic discharge after 1, 2, and 3 days. Float tests were performed by charging the PTMA‐based electrodes to 1.1 V vs. Ag at 1 C and keeping this voltage for a total of 220 h.

## Conflict of interest

The authors declare no conflict of interest.

## Supporting information

As a service to our authors and readers, this journal provides supporting information supplied by the authors. Such materials are peer reviewed and may be re‐organized for online delivery, but are not copy‐edited or typeset. Technical support issues arising from supporting information (other than missing files) should be addressed to the authors.

Supplementary
