# Supplementary material for: Emulsion Polymerizations for a Sustainable Preparation of Efficient TEMPO‐based Electrodes
Source: ChemSusChem. 2020 Nov 20;14(1):449–55. doi: 10.1002/cssc.202002251 (PMC7839472; doi:10.1002/cssc.202002251)
Supplement: Supplementary file 1 — Supplementary [file CSSC-14-449-s001.pdf]

ChemSusChem

Supporting Information

Emulsion Polymerizations for a Sustainable Preparation of Efficient TEMPO-based Electrodes

Simon Muench, Patrick Gerlach, René Burges, Maria Strumpf, Stephanie Hoeppener, Andreas Wild, Alexandra Lex-Balducci, Andrea Balducci, Johannes C. Brendel, and Ulrich S. Schubert*© 2020 The Authors. ChemSusChem published by Wiley-VCH GmbH. This is an open access article under the terms of the Creative Commons Attribution License, which permits use, distribution and reproduction in any medium, provided the original work is properly cited.

Supporting information

Emulsion polymerizations for a sustainable preparation of efficient TEMPO-based electrodes

Simon Muench,[a,b] Patrick Gerlach,[b,c] René Burges,[a,b] Maria Strumpf,[a,b] Stephanie Hoeppener,[a,d] Andreas Wild,[e] Alexandra Lex-Balducci,[a,b] Andrea Balducci,[b,c] Johannes C. Brendel,[a,b] and Ulrich S. Schubert[a,b]*

[a] Laboratory of Organic and Macromolecular Chemistry (IOMC)
Friedrich Schiller University Jena, Humboldtstr. 10, 07743 Jena, Germany
E-mail: ulrich.schubert@uni-jena.de, Homepage: www.schubert-group.de

[b] Center for Energy and Environmental Chemistry Jena (CEEC Jena)
Friedrich Schiller University Jena, Philosophenweg 7a, 07743 Jena, Germany

[c] Institute for Technical Chemistry and Environmental Chemistry
Friedrich Schiller University Jena, Philosophenweg 7a, 07743 Jena, Germany

[d] Jena Center for Soft Matter (JCSM)
Friedrich Schiller University Jena, Philosophenweg 7, 07743 Jena, Germany

[e] Evonik Operations GmbH | Research, Development & Innovation
Paul-Baumann-Straße 1, 45772 Marl, Germany

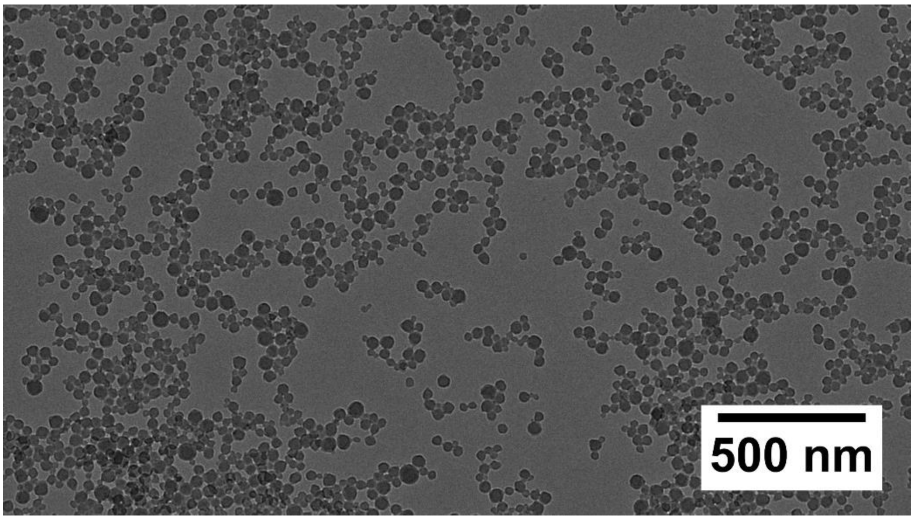

Figure S1: TEM image of poly(TMPMA) particles polymerized without cross-linker and with 3.6 mol% SDS; resulting particle size: 31±9 nm.

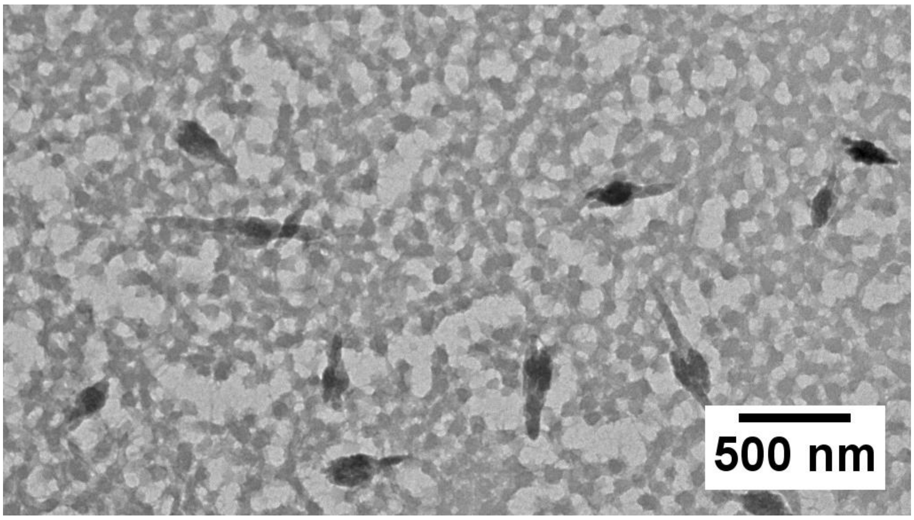

Figure S2: TEM image of poly(TMPMA) particles polymerized with 3 mol% TEG-DMA cross-linker and 3.6 mol% SDS.

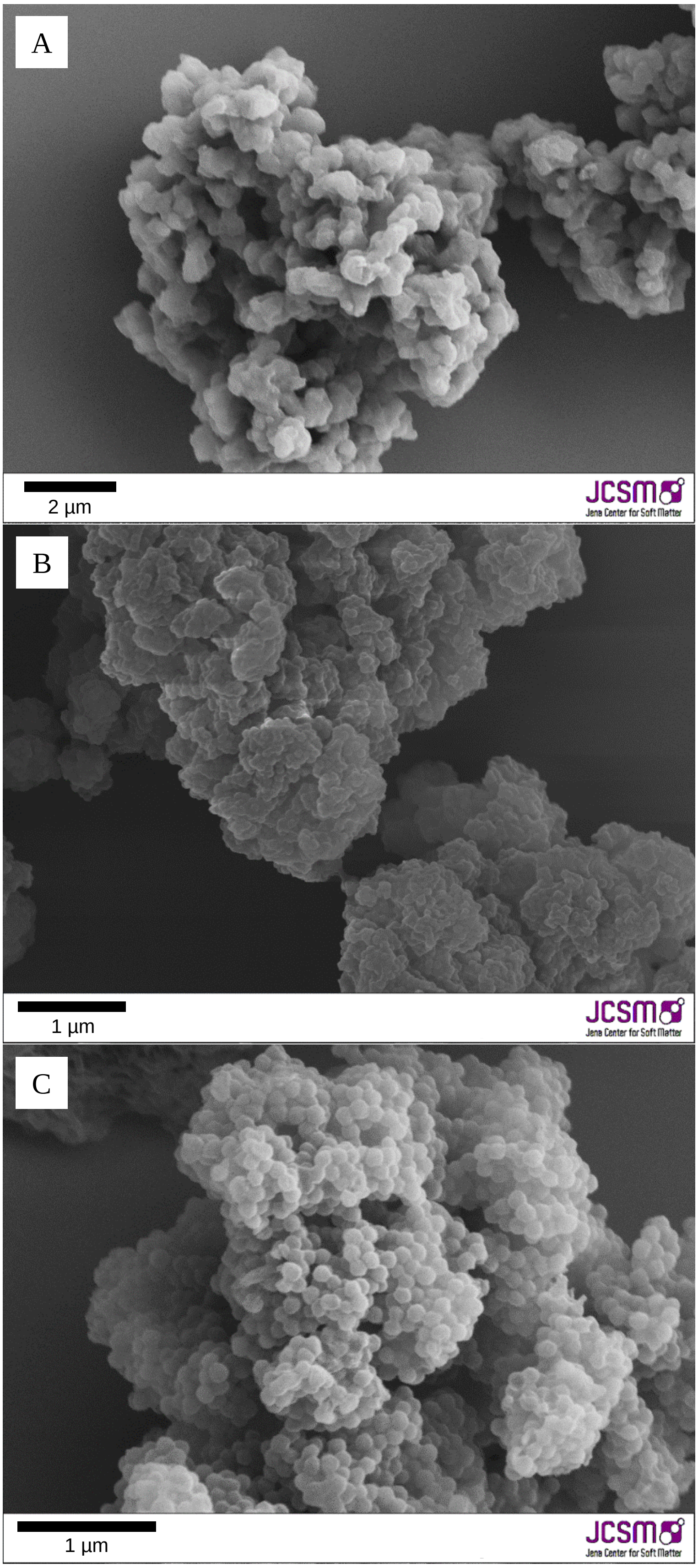

Figure S3: SEM images of particles after oxidation to PTMA and drying:
A) P1, B) P2, and C) P3.

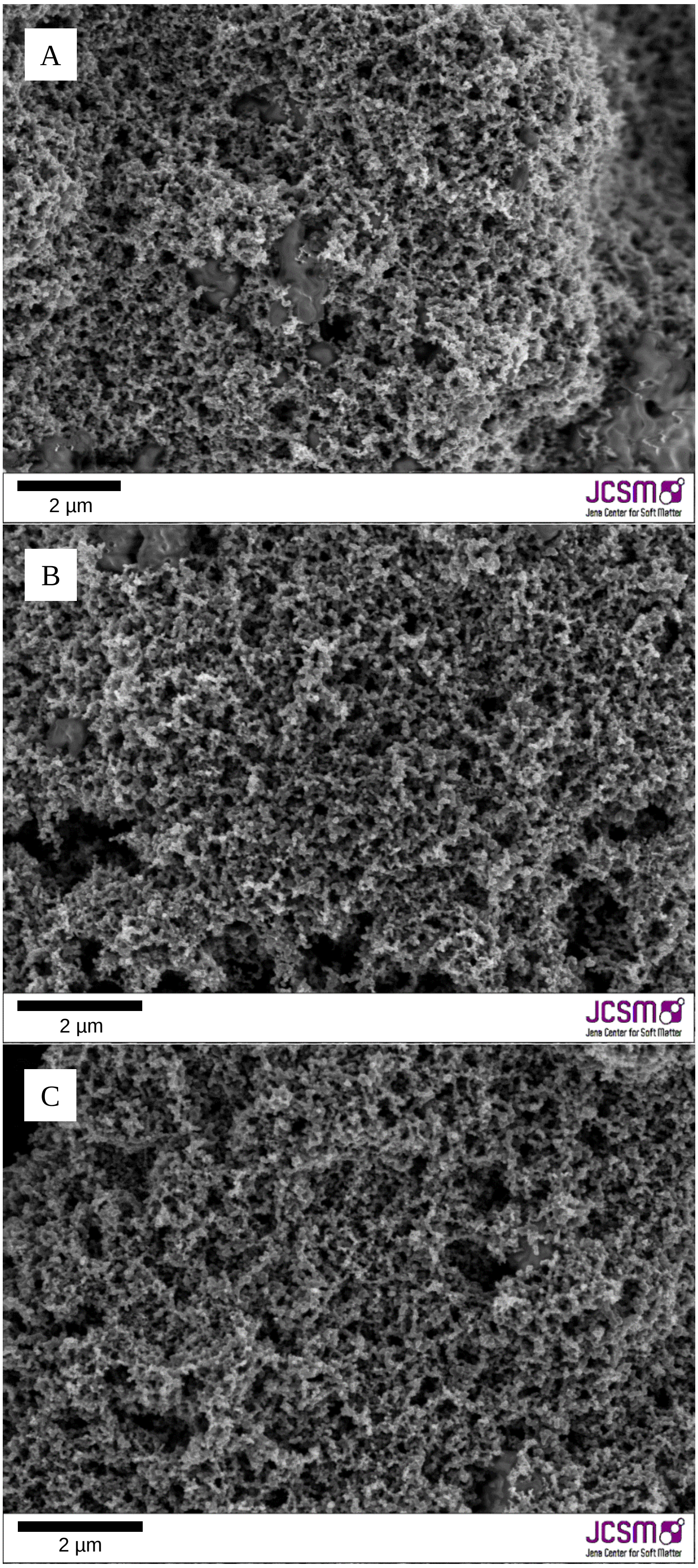

Figure S4: SEM images of composite electrodes containing the PTMA particles A) P1, B) P2, and C) P3.
